# Antioxidant Compounds, Kirenol and Methyl ent-16α, 17-dihydroxy-kauran-19-oate Bioactivity-Guided Isolated from *Siegesbeckia glabrescens* Attenuates MITF-Mediated Melanogenesis via Inhibition of Intracellular ROS Production

**DOI:** 10.3390/molecules26071940

**Published:** 2021-03-30

**Authors:** Sun-Yup Shim, Ye Eun Lee, Mina Lee

**Affiliations:** 1 Department of Food Science and Biotechnology, Sunchon National University, 255 Jungangno, Suncheon-si 57922, Korea; shimsy@scnu.ac.kr; 2 College of Pharmacy and Research Institute of Life and Pharmaceutical Sciences, Sunchon National University, 255 Jungangno, Suncheon-si 57922, Korea; qjsro1124@naver.com; 3 Institute of Jinan Red Ginseng, 41 Hongsamhanbang-Ro, Jinan-Eup, Jinan-Gun 55442, Korea

**Keywords:** *Siegesbeckia glabrescens*, kirenol, methyl ent-16α, 17-dihydroxy-kauran-19-oate, reactive oxygen species, melanin, microphthalmia-associated transcription factor

## Abstract

*Siegesbeckia glabrescens* (Compositae), an annual herb indigenous to Korean mountainous regions and has been eaten as a food in Korea. This study investigated ABTS, DPPH and nitric oxide (NO) radical-scavenging activities, and melanin production and TYR inhibitory effects-guided fractionation to identify therapeutic phytochemicals from *S. glabrescens* that can attenuate oxidation and melanogenesis in murine melanoma B16F10 cells. Nine compounds with inhibitory effects on melanin production, and TYR activity, and ABTS, DPPH, and NO radical scavenging activity were isolated from the 100% ethanol fraction from *S. glabrescens*. Among the nine compounds, kirenol (K), methyl ent-16α, 17-dihydroxy-kauran-19-oate (MDK) had strong inhibitory effects on melanin production and TYR activity with antioxidant effects. Western blot analysis revealed that K and MDK suppressed tyrosinase-related protein (TYRP)-1, TYRP-2 and microphthalmia-associated transcription factor (MITF) expression. Moreover, these two compounds inhibited intracellular reactive oxygen species (ROS) level in tert-butyl hydroperoxide (*t*-BHP)-treated B16F10 cells. Our results suggest that *S. glabrescens* containing active compounds such as K and MDK, which has antioxidant and antimelanogenesis effects, is the potent therapeutic and functional material for the prevention of oxidation-induced hyperpigmentation.

## 1. Introduction

Oxidative stress, which is defined as the generation of reactive oxygen species (ROS) and toxic free-radical species creates an imbalance between antioxidant and prooxidant homeostasis and is associated with the progression of various diseases [1]. ROS is constantly generated in the body from internal metabolism and external exposure, and it’s production has been detected in various diseases and has been shown to have several roles. Skin color depends on the quantity, quality, and distribution of the melanin pigment [2]. Exposure to environmental factors involving ultraviolet (UV) irradiation and various physical and chemical stimulation triggers skin ageing, thereby causing skin dryness, wrinkles, and hyperpigmentation [3,4]. Hyperpigmentation is related to melanin generation which is produced by melanocytes that are surrounded by keratinocytes and is a major factor that determines skin color and one of the defense systems that protects the skin from UV-induced skin damage. Melanoblasts are unpigmented precursor cells of melanocytes [5,6]. These cells migrate to various regions of the body and develop into melanocytes, and are identified by the expression of melanocyte-specific markers such as tyrosinase (TYR), tyrosinase-related protein (TYRP)-1, TYRP-2, and L-3,4-dihydroxyphenylalanine (DOPA) chrome tautomerase. The primary function of melanocytes is the production of the melanin pigment. Hyperpigmentation is the over-production of melanin in different parts of the body and causes visible skin pigmentary diseases involving albinism, leukoplakia, melasma, freckles, moles, and lentigo. TYR hydrozylates tyrosine to 3,4-dihydroxyphenylalanin (DOPA) that is oxidized to DOPA quinone in melanosomes of melanocytes. TYRP-1 and TYRP-2 are present in the membrane of melanosomes and catalyze the eumelanin-producing reactions. TYRP-1 has a role in the activation and stabilization of TYR, melanosome synthesis, increased eumelanin: pheomelanin ratio, and protects against oxidative stress by mediating peroxidase activity [7,8,9,10,11]. TYRP-2 acts as a DOPA chrome tautomerase and catalyses the conversion of L-dopachrome, a red melanin precursor to colorless dihydroxyindole-2-carboxylic acid (DHICA). In the absence of TYRP-2, L-DOPA chrome is converted to dihydroxyindole (DHI), a toxic melanin precursor [8,12]. Melanin synthesis is managed by a microphthalmia-associated transcription factor (MITF) [13]. MITF is activated in response to various external stimuli by signaling mechanism involving cAMP response-binding protein (CREB), Wnt, glycogen synthase kinase (GSK) 3β, and mitogen-activated protein kinases (MAPK), and regulates the expression of melanogenic enzymes such as tyrosinase (TYR) [14]. Cellular melanin synthesis can be modulated by the catalytic activity of TYR and TYR expression.

*Siegesbeckia glabrescens* (Compositae), an annual herb, is well-known as ‘Hui-Chum’ or ‘Hui-Ryeom’ in Korea and has been eaten as a food in Korea and its herbs are used as seasoned vegetable, called to ‘Namul’. It has also used as an oriental medicine for centuries to treat hypertension, osteoporotic fractures, quadriplegia, paralysis, and hemiplegia and the young leaves are used as food in Korea. In addition, this plant has been known to possess anti-angiogenic, anti-rheumatic, anti-inflammatory, anti-tumor, antiasthma, anticancer, antibacterial, and anti-ageing properties [15,16,17,18,19,20]. However, until date, there exist no reports on the inhibitory effects and the mechanism of action of *S. glabrescens* on melanogenesis. Antioxidants with antimelanogenesis effects are potentially useful for the attenuation of skin hyperpigmention disorders. Various natural-derived compounds have been reported to attenuate cellular melanin synthesis and many studies have focused on the discovery of novel natural skin-whitening materials that are currently in progress. In this study, anti-oxidant and anti-melanogenic compounds were obtained by inhibition of ABTS, DPPH and NO radical scavenging activity and melanin production and TYR activity-guided isolation from *S*. *glabrescens*, and the down-regulation of MITF-mediated TYR expression of its compounds with anti-oxidant effects were investigated.

## 2. Results and Discussion

### 2.1. Anti-Melanogenesis and Anti-Oxidant Effects of Extract

**Figure 1:**
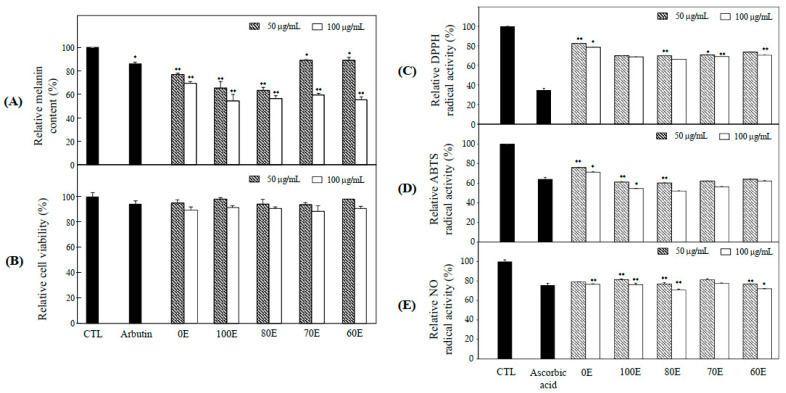
Anti-melanogenesis and anti-oxidant effects of *S. glabrescens* extracts. (**A**) Melanin content, (**B**) cytotoxicity in B16F10 cells, (**C**) DPPH and (**D**) ABTS, and (**E**) NO radical scavenging activities. The data are represented as the mean ± SD (n = 3) of three individual experiments. * *p* < 0.05, and ** *p* < 0.01 compared with control.

Hyperpigmentation disorders of skin have a severe impact on the quality of life and result from an increased generation of melanin in melanocytes [18]. Recently, the demand for natural hypopigmenting agents and cosmetics has rapidly increased because of oxidative damage [19]. Natural products as sources of bioactive compounds have been a subject of focus for the prevention of oxidative damage and the development of hypopigmenting agents. Melanin synthesis includes oxidation reaction, and suppression of oxidative damage is important in the protection of melanogenesis [18,19]. B16F10 cells, which is widely used for research as a model of hyperpigmentation as well as skin cancer, are derived from murine melanoma, and have all the elements necessary for melanin synthesis in the response to the oxidative stress [20]. Our study assessed the application of *S. glabrescens*, which is used as a food material on ABTS, DPPH and NO radical scavenging activities and melanin production and TYR activity-guided fractionation to discover natural therapeutic products that can reduce hyperpigmentation. Also, we investigated the optimal conditions of extraction regarding the anti-oxidant effects (ABTS, DPPH, and NO radical scavenging activities) and anti-melanogenesis effects (TYR activity and melanin production). For sample preparation, the dried form of *S. glabrescens* was extracted with 100%, 80%, 70%, and 60% ethanol. It was observed that the extracts suppressed melanin production at concentrations of 50 and 100 μg/mL. Among the extracts, 100% EOH extract (100E) strongly inhibited 45.7% of melanin production (Figure 1A). Compared to arbutin (500 μg/mL) which was used as a positive control, 100E of *S. glabrescens* extensively inhibited 31.5% of melanin production in B16F10 cells. The effects of the extracts on the cell viability of B16F10 cells were assessed using MTT assay to determine the non-cytotoxic concentration of the extracts. None of the extracts at concentrations of 50 and 100 μg/mL demonstrated cytotoxicity on B16F10 cells after treatment for 72 h (Figure 1B). Radical scavenging system involving DPPH, ABTS, and NO assays are excellent tools to investigate the antioxidant capabilities and mainly associated with the hydrogen donating or proton radical scavenging capabilities of target natural compounds [21]. We attempted that DPPH, ABTS and NO radical scavenging assays were conducted to assess the antioxidant effects of extracts of *S. glabrescens*. All the extracts shown antioxidant activity such as DPPH (Figure 1C), ABTS (Figure 1D) and NO (Figure 1E) radical scavenging activities. Accordingly, 100E of *S. glabrescens* with potent inhibitory activity of melanin production was used for subsequent experiments.

### 2.2. Bioactivity–Guided Isolation of Active Phytochemicals from S. glabrescens

**Figure 2:**
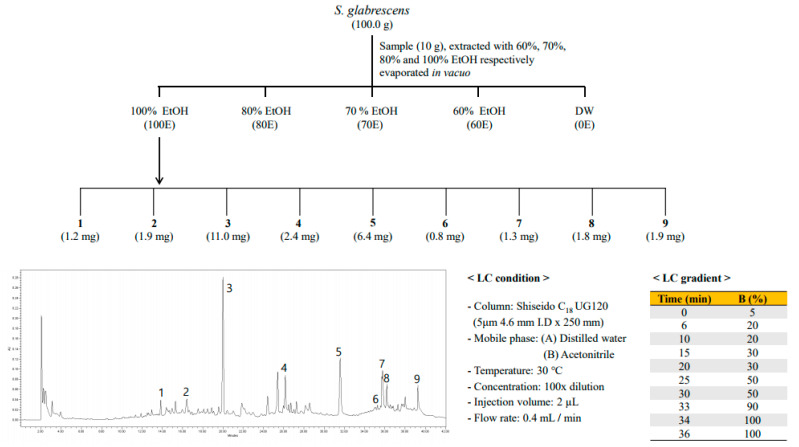
Schematic representation of the isolation of compounds **1** (K) and **9** (MDK) from *S. glabrescens* extract using bioactivity-guided fractionation. Bioactivity-guided fractionation of *S. glabrescens* was performed as shown in the schematic representation and resulted in the isolation and identification of compounds **1** and **9**. Fractionation was guided by assessing the inhibitory effects of K and MDK on melanin content and tyrosinase activity without cytotoxicity at 50 and 100 μg/mL. At each level of fractionation, all the fractions generated were tested simultaneously and were compared with the crude extract.

**Figure 3:**
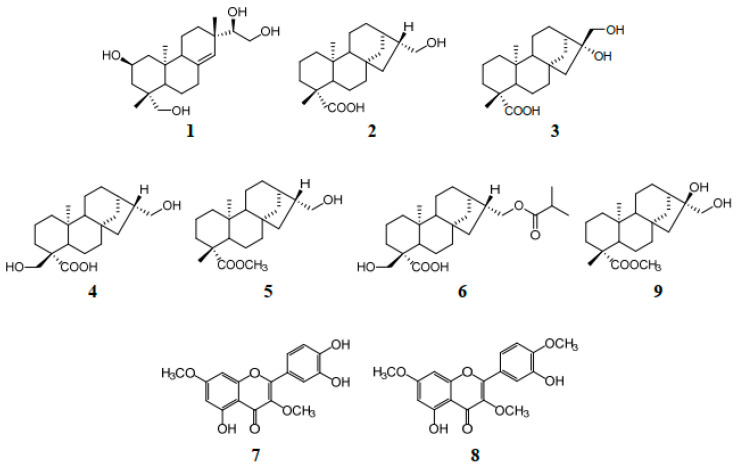
Chemical structures of compounds isolated from *S. glabrescens*.

Among the five extracts, 100E of *S. glabrescens* demonstrated a potent inhibitory effect on melanin production with DPPH, ABTS and NO radical scavenging activities, and was used to isolate the bioactive phytochemicals according to bioactivity-guided fractionation. Nine compounds were isolated by RP HPLC (Figure 2). The compounds were identified as kirenol (**1**), siegeskaurolic acid (**2**), ent-16α, 17-dihydroxy-kauran-19-oic-acid (**3**), Siegesbeckic acid (**4**), methyl ent-16αH-17-hydroxy-kauran-19-oate (**5**), enti-16βH, 17-isobutyryloxy-18-hydroxy-kaurane-19-oic acid (**6**), quercetin 3,7-*O*-dimethyl ether (**7**), quercetin 3,7,4′-trimethyl ether (**8**), methyl ent-16α, and 17-dihydroxy-kauran-19-oate (**9**) by comparison of the measured spectroscopic (NMR and MS) data with published data (Figure 3) [22,23,24,25,26,27]. Compounds **1**–**6** and **9** were diterpenoids and compounds **7** and **8** were flavonoids.

### 2.3. Anti-Melanogenesis and Anti-Oxidant Effects of Nine Compounds Isolated from S. glabrescens

**Figure 4:**
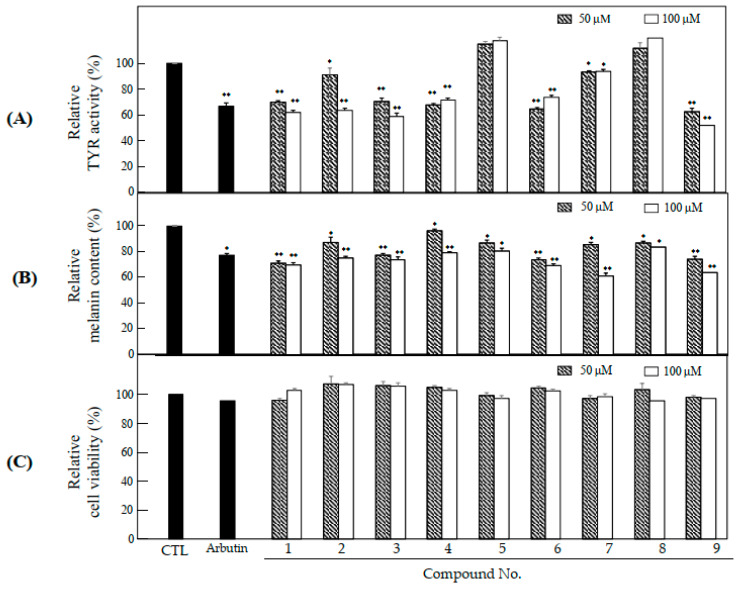

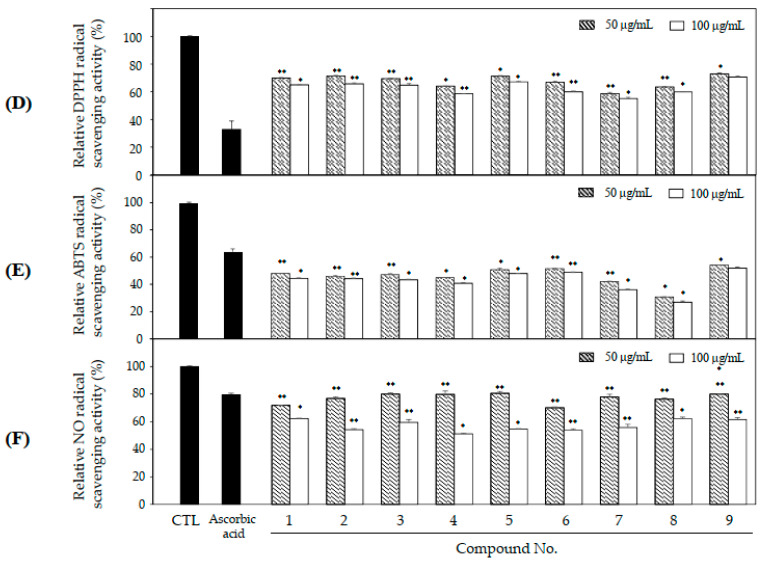
Anti-melanogenesis and anti-oxidant effects of nine compounds isolated from 100 E-OH fraction of *S. glabrescens*. (**A**) TYR activity, (**B**) melanin content, and (**C**) cytotoxicity in B16F10 cells, (**D**) DPPH and (**E**) ABTS, and (**F**) NO radical scavenging activities. The data are represented as the mean ± SD (n = 3) of three individual experiments. * *p* < 0.05 and ** *p* < 0.01 compared with control.

The nine compounds isolated from 100E of *S. glabrescens* herbs were investigated for their anti-melanogenesis and antioxidant effects. Among the nine compounds, compounds **1**, **3** and **9** at 100 μM inhibited the TYR activity by 37.8%, 41.2%, and 48.1%, respectively (Figure 4A). Also, melanin production in the presence of 100 μM of compounds **1**, **6**, and **9** was inhibited by 29.0%, 26.1%, and 25.9%, respectively (Figure 4B). These compounds showed higher inhibitory activity compared to the positive control, arbutin (500 μg/mL). All the nine compounds did not exhibit cytotoxicity at 50 and 100 μM in B16F10 cells (Figure 4C). Nine compounds were assessed antioxidant effects involving DPPH, ABTS, and NO radical scavenging activities. These compounds exhibited DPPH (Figure 4D), ABTS (Figure 4E), and NO (Figure 4F) radical scavenging activities withoud big difference. Among the nine compounds, kirenol (**1**; K), pimarane type diterpenoid, methyl ent-16α, 17-dihydroxy-kauran-19-oate (**9**; MDK) with antioxidant effects, and kaurene type diterpenoid demonstrated potent inhibitory activity on melanin production and TYR activity without mediating any cytotoxicity and were selected for the assessment of anti-melanogenesis effect.

### 2.4. Effects of K and MDK on Intracellular ROS Levels

**Figure 5:**
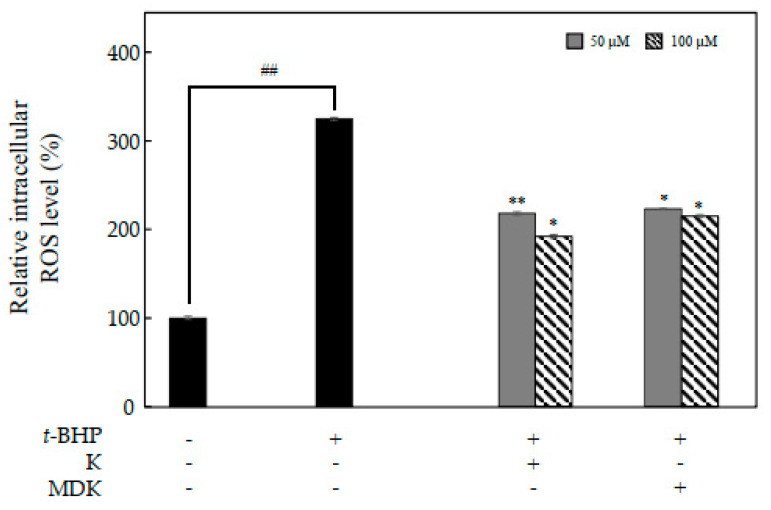
Effects of K and MDK on intracellular ROS generation in *t*-BHP-treated B16F10 cells. The data are represented as the mean ± SD (n = 3) of three individual experiments. ^##^ *p* < 0.01 vs. non-treated group; * *p* < 0.05 and ** *p* < 0.01 vs. *t*-BHP-treated group.

Melanin production involves oxidation reaction and is important for ROS generation and hyperpigmentation [5,6]. To investigate the inhibitory effects of 50 and 100 μM of K and MDK, intracellular ROS levels in *t*-BHP-treated B16F10 cells were examined using ROS specific probe, DCFH-DA (Figure 5). Oxidative stress is the major cause of ROS generation and induces disruption of melanocytes homeostasis, thereby resulting in melanin synthesis. Moreover, it is defined as the production of toxic free radical species and disturbance between anti-oxidant and pro-oxidant balance [6,28]. To investigate the correlation between the inhibition of hypopigmentation and antioxidant activities, intracellular ROS generation in melanoma cells by K and MDK was examined. As shown in Figure 5, K and MDK inhibited intracellular ROS production in B16F10 cells. Kirenol inhibited antioxidant enzyme activities such as SOD, CAT, GSH and increased ROS production for treatment of cancer cells through cancer signaling mechanism [29,30]. Our results suggested that K and MDK mediated inhibition of intracellular ROS level may be due to the radical scavenging activity of the compounds.

### 2.5. Effects of K and MDK on TYRP-1, TYRP-2, and MITF Expression

**Figure 6:**
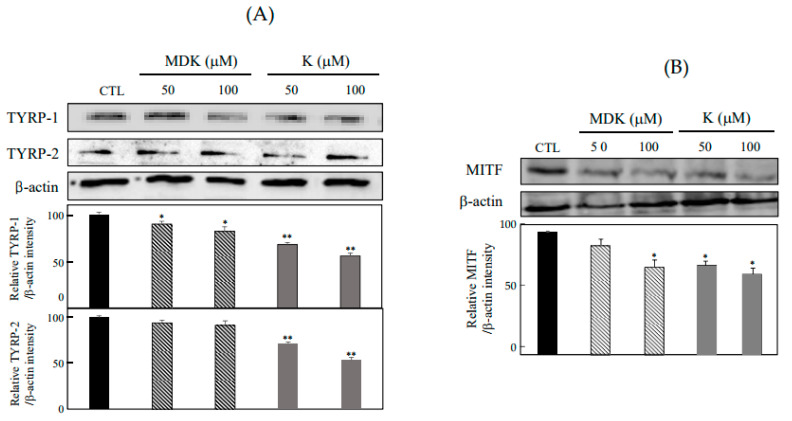
Effects of K and MDK on TYRP-1, TYRP-2 and MITF expression. The density graph represents the relative band density for TYRP-1, TYRP-2 (**A**) and MITF (**B**) normalized to β-actin. The data are represented as the mean ± SD (n = 3) of three individual experiments. * *p* < 0.05 and ** *p* < 0.01 compared with control.

TYRP-1 and TYRP-2 play an important role in determining the quality and quantity of melanin [11]. Melanin synthesis can be attenuated by the down-regulation of TYRP expression. To assess the protein inhibitory expression of TYRP-1 and TYRP-2 by K and MDK, Western blot assay was attempted using the specific antibodies. As shown in Figure 6, K and MDK inhibited the expression of these proteins at 50 and 100 μM. TYR protein family consists of TYR, TYRP-1, and TYRP-2. TYRP-1 is a melanocyte-specific gene product involved in generating melanin, stabilizing the TYR protein and regulating its catalytic activity, maintaining the melanosome structure, and influences melanocyte proliferation and melanocyte cell death. TYRP-2 is expressed very early during melanoblast differentiation and acts as a L-DOPA chrome to generate DHICA through eumelanin synthesis [31,32,33]. Our results showed that K and MDK inhibited TYRP-1 and TYRP-2 protein expression. Further studies on K and MDK mediated gene expression and down-regulation of TYRP-1 and TYRP-2 are necessitated to confirm the inhibitory relationship between melanin production and their proteins.

MITF, which regulates melanogenesis and dendricity in melanogenic signaling plays a pivotal role in the expression of melanogenic enzymes such as TYR, TYRP-1, and TYRP-2, and is a key transcription factor and essential regulator of proliferation, differentiation, and survival of melanocytes [7,32,33,34,35]. To confirm the inhibitory effects of K and MDK activation on MITF expression, Western blot analysis was performed using specific antibodies. As shown in Figure 6B, K and MDK down-regulated MITF expression at concentrations of 50 and 100 μM. We examined the inhibitory mechanism of K and MDK on melanogenesis. Our results revealed a decrease in melanin production and demonstrated that reduction was related to the expression of TYR (Figure 4A) and TYRP (Figure 6A), which is regulated by MITF. Moreover, it was evident that the anti-melanogenesis activity of K and MDK was related to the anti-oxidant activity such as inhibition of intracellular ROS production at the cellular level and radical scavenging activity in vitro (Figure 5). In the response to intracellular ROS production which is caused by oxidative stress, the MITF transcription factor drives the expression of a number of transcription factors including protein kinase C (PKC), cyclic adenosine monophosphate (cAMP), MAPK, CREB, phosphatidylinositol-4,5,-biphosphate (PI3K), protein kinase B (PKB/Akt), and glycogen synthase kinase (GSK) 3β and melanocyte-specific melanocortin-1 receptor (MCIR) [7,36,37].

**Figure 7:**
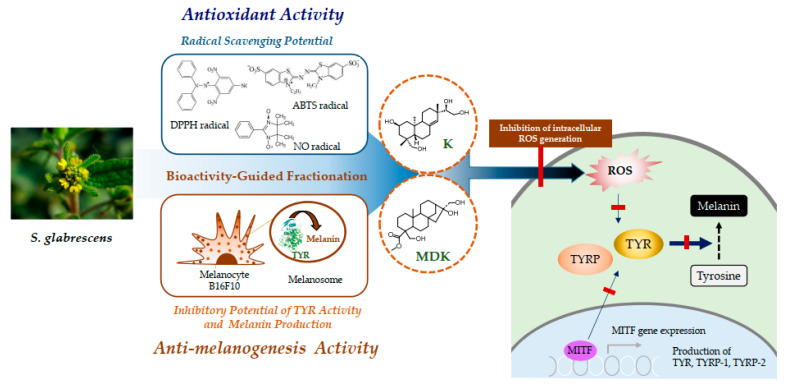
Summary on antimelanogenesis and antioxidant effects K and MDK isolated from *S. glabrescens* by bioactivity-guided fractionation.

Our results shown that K and MDK were isolated from *S. glabrescens* by antioxidant assay (DPPH, ABTS, and NO radical scavenging assay) and antimelanogenesis assay (TYR activity and melanin production). These compounds inhibited intracellular ROS production that caused down-regulation of MITF-mediated TYR, TYRP-1 and TYRP-2 expression (Figure 7). Further studies on the regulation of signaling transcriptional mechanism of K and MDK in melanocytes are necessary to confirm their potential therapeutic application in the protection of hyperpigmentation.

## 3. Materials and Methods

### 3.1. Plant Materials

*S. glabrescens* (Compositae) were purchased from the Kyungdong market in Seoul, Korea in September 2019 and identified by Mina Lee at Sunchon National University. A voucher specimen (SCNUP 24) was deposited in the Laboratory of Pharmacognosy, College of Pharmacy, Sunchon National University, Suncheon-si, Jeollanam-do, Korea.

### 3.2. Extraction and Isolation

The dried herbs (100 g) of *S. glabrescens* were pulverized and sonicated 3 times for 2 h with 100%, 80%, 70%, and 60% EtOH and 100% distilled water, respectively. The extracts solution was concentrated and used to measure the cell viability and bioactivities in murine melanoma B16F10 cells. Among them, the 100% EtOH extract with most potent anti-melanogenesis effect but no cytotoxicity was dissolved with 100% MeOH and separated by column chromatography. Compound **1** (1.2 mg, tR 14.61 min), **2** (1.9 mg, tR 16.22 min), **3** (11.0 mg, tR 19.75 min), **4** (2.4 mg, tR 21.06 min), **5** (6.4 mg, tR 31.36 min), **6** (0.8 mg, tR 35.12 min), **7** (1.3 mg, tR 35.68 min), **8** (1.8 mg, tR 36.11 min), and **9** (1.9 mg, tR 39.18 min) were obtained by separation using a multi-gradient reverse phase (RP) high-performance liquid chromatography (HPLC) system (Shiseido UG120, C_18_, 250 × 4.6 mm, H_2_O:CH_3_CN = 95:5 → 100% CH_3_CN, 0.4 mL/min).

Kirenol (K) (**1**). C_20_H_34_O_4_; white amorphous powder; ^13^C NMR (100 MHz, DMSO-*d*_6_): δ 138.0 (C-8), 129.9 (C-14), 76.6 (C-15), 64.8 (C-19), 64.0 (C-16), 63.9 (C-2), 55.5 (C-5), 51.3 (C-9), 49.4 (C-1), 45.7 (C-3), 41.0 (C-4), 39.7 (C-10), 38.0 (C-13), 36.8 (C-7), 32.8 (C-12), 28.3 (C-18), 23.2 (C-17), 22.6 (C-6), 19.0 (C-11), 17.0 (C-20). Electrospray ionization mass spectrometry (ESIMS) *m/z* 337 [M − H]^−^.

Methyl ent-16α, 17-dihydroxy-kauran-19-oate (MDK) (**9**). C_21_H_34_O_4_; white amorphous powder; ^13^C NMR (100 MHz, DMSO-*d*_6_): δ 178.1 (C-19), 82.0 (C-16), 66.8 (C-17), 57.2 (C-5), 56.5 (C-9), 54.1 (C-15), 51.4 (C-21), 46.2 (C-13), 45.2 (C-8), 44.3 (C-4), 42.9 (C-7), 41.1 (C-1), 40.0 (C-10), 38.7 (C-3), 38.1 (C-14), 29.0 (C-18), 27.1 (C-12), 23.0 (C-6), 19.9 (C-2), 29.3 (C-11), 15.9 (C-20). ESIMS *m/z* 349 [M − H]^−^.

### 3.3. Cell Culture and Viability Assay

B16F10 mouse melanoma cells were purchased from the Korean Cell Line Bank (KCLB) (Seoul, Korea). The cells were cultured at 37 °C in high glucose DMEM (HyClone, Logan, UT, USA), supplemented with 10% fetal bovine serum (FBS) (HyClone, Logan, UT, USA), 100 IU/mL of penicillin, and 100 mg/mL of streptomycin (HyClone, Logan, UT, USA) in a humidified atmosphere containing 95% air and 5% CO_2_. The extracts, each fraction, and compounds were dissolved in dimethylsulfoxide (DMSO) (Sigma) for anti-melanogenesis assay in B16F10 cells. The cytotoxicity of *S. glabrescens* was assessed by quantifying the cell viabilities in the presence of each sample. The cells (1 × 10^4^ cells/well) were seeded and incubated for 24 h in DMEM supplemented with 10% FBS. The culture medium was replaced with serum-free DMEM and treated with the compounds at different concentrations for 72 h. After removing the serum-free DMEM, the cells were treated with MTT solution (0.5 mg/mL) and incubated for 4 h. After replacing the medium with DMSO, the number of live cells was measured at 570 nm on a microplate reader (BioTek Instruments, Winooski, VT, USA).

### 3.4. Melanin Content Assay

The intracellular melanin content was determined using a slightly modified procedure described elsewhere [38]. Briefly, B16F10 cells were seeded in a 24-well plate at 2 × 10^4^ cells/well and incubated for 24 h in DMEM containing 10% FBS. The cells were pretreated with the sample for 72 h, washed with PBS, and dissolved in 1N NaOH containing 10% DMSO by boiling at 80 °C for 1 h. The cell lysates were centrifuged at 14,000 rpm for 10 min. The absorbance of the supernatant was measured at 490 nm. The melanin content is expressed as a percentage of the control. Arbutin (500 μg/mL) was used as a positive control.

### 3.5. Cellular TYR Activity Assay

The cellular TYR activity was measured as the L-DOPA oxidase activity using a slightly modified method described previously [39,40]. The B16F10 cells were seeded and incubated for 24 h in a 24 well plate in the presence or absence of the samples for 72 h. After treatment, the cells were washed with cold PBS and lysed with a lysis buffer (0.1 M sodium phosphate buffer, 1% Triton X-100 and 0.1 mM PMSF). The cell lysates were centrifuged at 10,000 rpm for 10 min. The supernatant was used as the cellular TYR solution. The reaction mixture containing 40 μL of cell lysate and 100 μL of L-DOPA (2 mg/mL) (Sigma-Aldrich, Co., St. Louis, MO, USA) was incubated at 37 °C for 1 h and the level of dopachrome formation was measured spectrophotometrically at 490 nm. RIPA buffer was used as the control. The TYR activity was calculated as a percentage of the control; arbutin (500 μg/mL) was used as the positive control.

### 3.6. Intracellular ROS Assay

Intracellular ROS production was assessed with an oxidant-sensitive fluorescent probe, DCFH-DA. B16F10 melanoma cells were seeded at 1 × 10^4^ cells/well and incubated with various concentrations of K and MDK for 24 h. The cells were treated with tert-butyl hydroperoxide (*t*-BHP) (Sigma-Aldrich, Co., St. Louis, MO, USA) (400 μM) for 2 h to induce ROS production and then incubated with 25 μM of DCFH-DA (Sigma-Aldrich, Co., St. Louis, MO, USA) for 30 min. The fluorescence intensities were measured at an excitation wavelength of 485 nm and an emission wavelength of 535 nm on a fluorescence microplate reader (Berthold Technologies GmbH & Co, KG US, Bad Wildbad, Germany).

### 3.7. In Vitro Antioxidant Assay

In vitro antioxidant assay of extracts and compounds was performed by 2,2-azino-bis (3-ethylbenzothiazoline-6-sulfonic acid diammonium salt (ABTS), 1,1-diphenyl-β-picrylhydrazine (DPPH) and nitric oxide (NO) radical scavenging activities.

#### 3.7.1. ABTS Assay

The ABTS (Sigma-Aldrich. Co) radical scavenging activity was measured using a previously reported method by Proestos with slight modification [41]. 2,2′-azobis (2-aminopropane) dihydrochloride (7 mM) (Sigma-Aldrich, Co., St. Louis, MO, USA) was mixed with 2.45 mM ABTS and allowed to react for 16 h at 4 °C. Typically, 50 μL of the sample and 100 μL of the ABTS solution were mixed and allowed to react at 23 °C for 20 min after addition to a 96 well plate. The absorbance was measured at 734 nm using a microplate spectrophotometer (Epoch, Biotek Instruments, Inc., VT, USA). Ascorbic acid (100 μg/mL) was used as a positive control.

#### 3.7.2. DPPH Radical Scavenging Assay

The radical scavenging effect of DPPH (Sigma-Aldrich, Co., St. Louis, MO, USA) was measured using the method reported elsewhere with a slight modification [42]. A 100 μL of DPPH solution (0.2 mM) was added to 100 μL of the sample on a 96 well plate, mixed for five seconds, and allowed to react for 30 min under shade. The absorbance was measured at 517 nm using a microplate spectrophotometer (Epoch, Biotek Instruments, Inc., VT, USA), ascorbic acid (100 μg/mL) (Sigma) was used as the positive control.

#### 3.7.3. NO Radical Scavenging Assay

The NO radical scavenging activity was measured using the method reported elsewhere with a slight modification [43]. A 180 μL of extract and compounds and 20 μL of 100 mM sodium nitroprusside (SNP) were mixed and incubated at RT for 150 min. The mixture was mixed with an equal volume of freshly prepared Griess reagent at RT for 15 min and then measured at 540 nm using a microplate spectrophotometer (Epoch, Biotek Instruments, Inc., VT, USA). Ascorbic acid (100 μg/mL) (Sigma) was used as the positive control.

### 3.8. Western Blot Analysis

The protein expression of TYRP-1, TYRP-2, and MITF was assessed by Western Blot analysis. B16F10 cells were treated with K and MDK at 50 and 100 μM for 72 h. The pretreated B16F10 cells were washed with cold PBS and harvested using trypsin-EDTA. The whole-cell lysates were extracted with a protein extraction kit (InTRON Biotechnology, Korea). Equal amounts of protein were separated by 10% SDS-PAGE and transferred to PVDF membranes (Merck Millipore, Darmstadt, Germany). The membrane was blocked with 5% skim milk in plain buffer (20 mM Tris pH 7.4 and 136 mM NaCl) at RT for 1 h, and incubated overnight with the primary antibodies against TYRP-1, TYRP-2, and MITF (Santa Cruz Biotech, CA, USA) at 4 °C. Subsequently, the membrane was incubated with a 500-fold dilution of specific secondary HRP-conjugated antibody (Thermo Fisher Sci., Waltham, MA, USA) at RT for 1 h, and the immunoreactive bands were visualized using an enhanced chemiluminescence ECL assay kit according to the manufacturer’s instructions.

### 3.9. Statistical Analysis

All measurements were conducted independently and at least in triplicate. Data are expressed as the mean ± SD. The significant differences between the control and the sample were determined using one-way ANOVA and Duncan’s multiple range tests using the software package SPSS statistics V20 at *p* < 0.05.

## 4. Conclusions

The 100E of *S. glabrescens* exhibited antioxidant and antimelanogenesis effects and nine compounds were isolated using bioactivity-guided fractionation. Among the nine compounds, K and MDK with antioxidant effects were exhibited potent inhibitory activity on melanin production and TYR activity, and were selected for further experiments. These two compounds exhibited inhibitory effects on TYRP-1, TYRP-2, MITF expression. Moreover, the compounds demonstrated anti-oxidative effects involving intracellular ROS production inhibition. Overall, it is hypothesized that *S. glabrescens* could be a potential therapeutic candidate and functional food against hyperpigmentation. K and MDK with antioxidant effects, the phytochemicals isolated from *S. glabrescens* suppressed melanin production via down-regulation of MITF-mediated TYR, TYRP-1 and TYRP-2 activation. Taken together, *S. glabrescens* containing potent antioxidant and antimelanogenesis compounds such as K and MDK could be useful functional materials for the protection of hyperpigmentation.

## Sample Availability

Samples of the compounds are available from the authors.
